# Cervical cancer: a meta-analysis, therapy and future of nanomedicine

**DOI:** 10.3332/ecancer.2020.1111

**Published:** 2020-09-24

**Authors:** Jeaneen Venkatas, Moganavelli Singh

**Affiliations:** Nano-Gene and Drug Delivery Group, Discipline of Biochemistry, School of Life Sciences, University of KwaZulu-Natal, Private Bag X54001, Durban 4000, South Africa; a https://orcid.org/0000-0001-5061-0788; b https://orcid.org/0000-0002-9985-6567

**Keywords:** cervical cancer, nanotechnology, nanomedicine, therapeutics, risk factors

## Abstract

Cervical cancer is one of the leading causes of female death, with an annual mortality rate exceeding 200,000 in developing communities. Despite the past decade bearing witness to a reduction in cervical cancer cases throughout developed countries, the prevalence in developing countries continues to rapidly rise. The increase in cervical cancer cases is attributed to the lack of financial resources and the unavoidable risk factors of the disease. Traditional means of anticancer therapy are compromised by reduced drug potency, non-specificity, negative side effects and the development of multiple drug resistance (MDR), which leads to a decrease in the long-term anticancer therapeutic efficacy. Recent advances in nanomedicine have elucidated the potential of nanoparticles to reduce the side effects and improve the survival rate of patients, by enhancing selective delivery and uptake of photosensitive, therapeutic and genetic material to cervical cancer cells, thereby enhancing antitumour efficiency. This review paper analyses the risk factors and epidemiology of cervical cancer globally, especially in developing communities, whilst demonstrating the enhanced anticancer treatment using selected nanoparticles.

## Introduction

Cancer produces one of the highest mortalities worldwide, with cervical cancer being the second most common malignancy amongst women. Cervical cancer is a growing health concern, with a global estimate of 570,000 novel cases and 311,000 deaths annually [1–3]. Despite the prevention of the disease by screening and treatment of pre-cancerous lesions, cervical cancer is the most common cause of cancer mortality amongst women [4]. The identification of key risk factors plays a fundamental role in cervical cancer prevention. Numerous studies have demonstrated the association between several risk factors and cancer [5–8].

An association between cervical cancer survival rate and the socioeconomic status of women has been reported [9]. In addition, venereal diseases, reproductive factors, long-term oral contraceptives and behavioural issues such as smoking and obesity have also been identified as risk factors for the disease [10]. High-risk human papillomavirus (HPV) infections have been established as the primary risk factor for the development of cervical cancer with HPV 16 and 18 being declared to be the cause of 71% of cervical cancer cases within the African continent [5].

Over the years, various treatment strategies have been developed for cervical cancer, including radiotherapy, chemotherapy and, in extreme cases, surgery [1]. However, these therapies are limited by a lack of anticancer drug potency, non-specificity, negative side effects and the development of MDR, which leads to a decrease in the long-term efficacy of anticancer therapy [11]. Nanotechnology has the potential to overcome these limitations, by increasing the selectivity and potency of chemical, physical and biological approaches for eliciting cancer cell death whilst minimising collateral toxicity to non-malignant cells [12, 13]. The favourable and unique properties of nanoparticles can improve the delivery of therapeutics, thereby enhancing their activity in cervical cancer cells whilst reducing harmful side effects in healthy cells [14, 15]. Hence, this meta-analysis aims to review the potential of nanomedicine in the improvement of cervical cancer therapeutics, especially in developing countries.

## Literature search criteria

To assess the potential of nanomedicine in cervical cancer therapeutics, several databases from the National Centre for Biotechnology Information, WorldCat.org and National Library of Medicine were searched from 2013 to March 2020 for studies throughout the world which were published in English. The literature was limited to case-controlled studies, randomised controlled trials and observational studies, with comparison groups addressing the incidence and mortality rate of cervical cancer in women aged 15–70, and risk factors including reproductive and behavioural factors and the economic status of a country. These databases were further searched to identify and compare both novel and traditional means of cervical cancer therapies. A total of 111 research articles that followed a consistent, precise and direct design, with minimised bias according to the PRISMA guidelines, were selected. The search terms were developed using a combination of the following keywords, cervical cancer, nanomedicine, nanoparticles, cervical cancer therapy, incidence and mortality rates, apoptotic pathways and risk factors.

## The burden of cervical cancer

Cancer cells modify the natural pathways of healthy cells enabling them to survive in harmful environments. These modified cells allow for metastasis and uncontrollable invasion of surrounding cells, inhibiting the normal function of fundamental organs. Cervical cancer results from a malignancy in the interior cervical lining and junction between the vagina and uterus [16]. Cervical cancer is primarily classified by histologic abnormalities, namely squamous cell carcinoma (SCC) and adenocarcinoma. The former accounts for 70%, whereas the latter accounts for 18% of cervical cancer cases. The remaining cases of cervical carcinomas consist of adenosquamous (4%) and additional carcinomas or malignancies (6.5%) [17, 18].

SCC develops within the transformation zone, located between cervical columnar and squamous cells, and migrates to the distal endocervical canal from the exocervix with an age-related progression [19]. Adenocarcinoma develops within the endocervix mucus glands [18]. Cervical cancer within the neuroendocrine is aggressive and often misdiagnosed and rare [20]. Cervical melanomas result from the migration of metastasised lesions from any other part of the body of all susceptible women, whereas cervical adenoid cystic carcinomas are mostly present in elderly women in the early stages of diagnosis [21]. Cervical lymphoma is another rare type of cervical cancer that occurs within the lymph nodes in the cervical area [22].

The development of cervical cancer is preceded by precancerous changes within the cervix. Precancerous lesions are referred to as squamous intraepithelial lesions (SIL) or cervical intraepithelial neoplasm (CIN). CIN is categorised into three broad categories, namely CIN1 (mild dysplasia), CIN2 (moderate dysplasia) and CIN3 (severe dysplasia) [23, 24]. It was reported that CIN1 and CIN2 were more likely to regress than progress, with a less than 1% progression rate from mild to severe dysplasia [25]. These results were confirmed by other researchers who also indicated that higher-grade lesions of CIN2 and CIN3 are more likely to progress to invasive carcinomas, with CIN3 displaying a progression rate of 31.1% [26].

The severity and site of infection denote the various stages of cervical cancer [27]. The initial stage, stage 0 or cervical carcinoma *in situ*, occurs within the upper cervical cell layer. Although stage 0 cervical cancer may develop malignancies if left untreated, it is not regarded by many as cancer [28]. Stage 1 is limited to the cervix only and is divided into two groups, stage 1A (5–7 mm) and 1B (>7 mm) with regards to the size of cancerous tissue. Cancer progresses from stage 1 to 2 when it begins to spread from the cervix into the upper vagina.

Similar to stage 1, stage 2 is divided into two categories, namely, stage 2A cervical cancer which spreads to about two-thirds of the vagina and stage 2B which progresses within the vagina to the surrounding tissues of the uterus [29]. Stage 3A cervical cancer spreads to the lower vagina and surrounding lymph nodes and is followed by stage 3B, whereby cervical cancer spreads to the pelvic wall. At this stage, the large size of tumour blocks the ureters causing the kidney to enlarge or even cease to function. Stage 4 is the most crucial stage, whereby cancer has the potential to spread to the rectum, bladder, intestinal tract and lungs [30, 31]. Cervical cancer prevention and control are targeted at the epidemiological studies describing the distribution and incidence of cervical cancer whilst accessing risk factors.

## Epidemiology of cervical cancer

### Global distribution and incidence rates of cervical cancer

**Figure 1.**
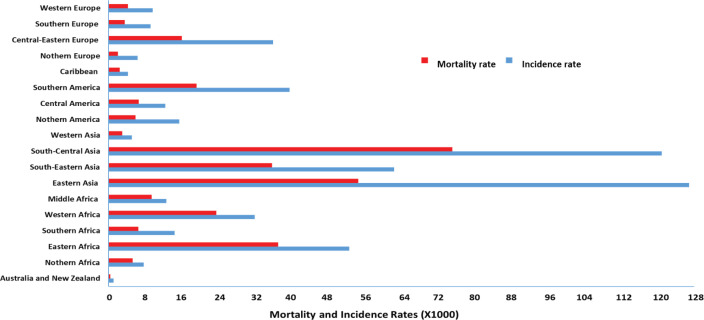
Global mortality and incidence rate of cervical cancer (per 100,000 individuals) [2, 34–38].

Cervical cancer is the second most common malignancy worldwide, with the incidence and mortality rate varying with geographical location (Figure 1) [2, 3]. Cervical cancer incidence and mortality rate in high-income countries have halved over the past 30 years due to the introduction of formalised screening programmes [30]. Sriplung *et al* [32] reported the incidence of cervical cancer across five continents. Although the authors postulated a decrease in the incidence rate in high-income countries, an increase in newly diagnosed cervical cancer cases was observed in low-income, developing countries.

Bray *et al* [2] depicted the inverse correlation between a country’s economic status and incidence of cervical cancer, whereas prevalence increased as economic status decreased. Ferlay *et al* [33] estimated that for every 50% of women screened in developed countries, only 5% are screened in developing countries. Arbyn *et al* [16] reported the lowest cervical cancer incidence and mortality rate to occur in high-income, developed countries. The authors stipulated the lowest incidence rates of cervical cancer (5,092 per 100,000) to occur in western Asia, whereas the lowest mortality rate was observed in Australia–New Zealand (403 per 100,000).

Eighty percent of cervical cancer cases occur in the developing world, in countries such as Switzerland (52/100,000) and Haiti (93/100,000) [36, 37]. The cervical cancer mortality rate in high-to-low human development index countries was reported to be 6.8 per 100,000 individuals [36] and a leading cause of incidence and mortality in Indian women with 122,844 novel cases and 67,477 deaths annually [38]. In 2015, approximately 98,900 new cases were reported in China, which accounted for 18.7% of the global incidence [39]. It was postulated that in rural China, which contains more than 70% of its population, 90% of women diagnosed with cervical cancer die within a 5-year period [40], making cervical cancer a major health concern in China.

Southern African countries bear a highly disproportionate incidence (92,000) and mortality (60,098) of cervical cancer [1, 2]. Similar results were reported revealing the incidence of cervical cancer to vary within Sub-Saharan Africa, with Malawi having the highest incidence of >72.9 per 100000 and mortality rates of 49.8 per 100,000 [41]. Conversely, in Nigeria, the cervical cancer incidence rate is 27.1/100,000 as a result of the National Cancer Control Policy implemented by the government to improve and control cancer, including cervical cancer [42].

The variation in socioeconomic status amongst different regions of the world plays a fundamental role in the development and implementation of screening programmes. An inadequate screening of cervical cancer in low-resource or developing countries is attributed to reduced health care due to financial constraints, lack of resources, inadequate knowledge of cervical cancer, healthcare infrastructure, laboratory supplies, trained practitioners and patient management guidelines and cultural and socioreligious barriers preventing routine pelvic screening [9, 43]. Like many cancers, the incidence and mortality rate discrepancies of cervical cancer in different regions are attributed to these risk factors.

### Cervical cancer risk factors

Epidemiological studies have identified several risk factors for cancer. Lifestyle factors such as reproduction (parity, pregnancy and contraceptives), obesity, diet, smoking and alcohol consumption have been included as major contributors to the development of cancer. Accessing the mechanism of each risk factor allows researchers to modify their intrinsic and extrinsic effects and effectively reduce the incidence of various cancers [10]. Cervical cancer prevalence begins to increase in women aged 18–29 years, peaks in women aged 48–64 and decreases in women older than 65 years [44].

#### Venereal diseases

Genetic factors play an essential role in the incidence of cervical cancer [35]. An important etiological factor for cancerous cervical lesions is a highly prevalent, sexually transmitted infection, HPV. HPV detection and vaccination measures need to be implemented as a means of cervical cancer prevention. Whilst 90% of HPV-induced lesions disappear within 6–12 months, others progress into cancer [35], with HPV types 16, 18, 45, 33, 35, 52, 51 and 31 associated with cervical cancer [45].

High-risk HPVs 16 and 18 account for 70% of novel cases globally [46], with a reported 71% of cervical cancer cases in Africa. The higher HPV prevalence in females compared to males has been reported to be due to a larger surface area within the epithelium of the cervix enduring squamous metaplasia and lack of certain adaptive immune responses observed in males. HPVs 16 and 18 induce carcinogenesis by oncoprotein E6 and E7 activities, which inhibit the function of *p53* and retinoblastoma tumour suppressor genes [5]. These oncoproteins are associated with changes in the methylation of both the host and viral DNA by altering cellular pathways that regulate cell adhesion, genetic integrity, cellular control, immune response and, most importantly, apoptosis [35, 47].

**Figure 2.**
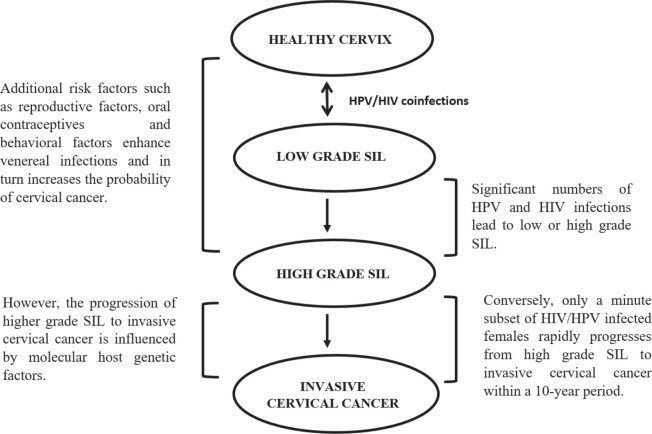
The influence of venereal diseases, HPV and HIV infections on cervical cancer development [5, 35, 47, 48, 52].

Cervical cancer forms a large portion of opportunistic diseases with an increased susceptibility of human immunodeficiency virus (HIV)-infected women to high-risk HPV-related cancers [36, 48]. HIV-1 increases the severity of lesions and the proportion of cervical cancer cases [49, 50], by attacking the CD4 or T-helper cells of the immune system and hijacking the cell’s replication machinery to replicate its own DNA. As HIV destroys more CD4 cells, the individual’s immune system becomes weak and unable to fight secondary diseases [51]. HIV may be directly involved in the oncogenesis or indirectly, due to immunosuppression [52]. HIV-positive females are at a higher risk of developing SIL which rapidly progresses to cervical cancer, which is aggravated in immunosuppressed individuals (Figure 2) [48]. HIV-1 increases the expression of HPV oncoproteins E6 and E7, which bind to and degrade the tumour suppressor proteins [49].

#### Reproductive factors

Cervical cancer is also linked to an increased susceptibility of HPV infections in individuals with multiple sexual partners [53]. A multivariate analysis model demonstrated that an earlier age of sexual debut and a higher parity is associated with a high-risk HPV infection [5]. Numerous studies have illustrated a direct correlation between parity and the prevalence of cervical cancer, whereby the age of a female’s first pregnancy is inversely associated with their susceptibility of cervical cancer [7, 8, 54]. Parity is regarded as the number of times an individual gives birth to a foetus within a 24-week gestational period [55]. It was illustrated that women who had a higher parity (greater than 3 pregnancies) were more likely to obtain invasive cervical cancer (odds ratio 2.1–2.5), compared to those with fewer pregnancies [8, 55]. It was further reported that delivery was a major predictor of CIN 3 and was significantly higher in women with HPV-infections and that parity influenced the quantity of oestrogen within a fertile woman, whereas oestradiol enhanced the immortalisation of HPV-infected cells [56].

Pregnancy may also produce some dysplastic lesions of the cervix, which may regress, persist or progress to produce a carcinoma [55, 57]. Despite the alarming evidence that parity is a predictor of cervical cancer, no positive relationship between parity and HPV has been established. Contradictory, the authors observed a higher parity in cervical cancer patients who were infected with HPV compared to cervical cancer patients who were not infected with HPV. However, it was suggested that the relationship between parity and cervical cancer is the result of sexual activity as a cofounding factor and may be eliminated with the use of contraceptives [58].

#### Oral contraceptives

From the late 1950s to the present day, billions of women have been utilising steroid hormones as a means of contraception. Oral contraceptives are synthetic hormone-containing drugs that are ingested to prevent pregnancy by inhibiting ovulation [54]. Several studies have depicted the strong correlation between cervical cancer and long-term oral contraceptive use [7, 54, 59, 60]. Naturally occurring oestrogen and progesterone have been observed to stimulate the growth and development of cancer cells. Hence, the introduction of additional synthetic hormones will accelerate cell growth and increase the risk of cancer [59]. It has been reported that the incidence of cervical cancer doubled in individuals who had consumed steroid hormones for longer than 5 years, with an increase in invasive cervical cancer prevalence in individuals taking injectable progesterone [35, 57]. The additional hormones increase the incidence of cervical cancer by increasing the susceptibility of high-risk HPV infections [59]. However, despite the increased incidence of unusual histological types in women who are long-term contraceptives users, the role of oral contraceptives in cervical cancer is still controversial [7, 60]. Hence, further studies on this association need to be undertaken.

#### Behavioural factors

The link between smoking and cervical cancer has been well elucidated, with smoking influencing the incidence of CIN 3 and invasive cervical cancer [6, 54, 61]. This positive correlation is due to the direct mutagenic effect of nicotine (decomposed product of cigarettes) on the DNA in squamous cells. Smoking also causes epigenetic changes within the epithelial cells contributing to the pathogenesis of cervical neoplasia [62]. Hence, smoker females are 2–4 times more susceptible to developing cervical cancer than non-smokers [57, 60]. It was also reported that the incidence of cervical cancer decreased dramatically (50%) in individuals who quit smoking for a 10-year period [54]. Smoking reduces the efficiency of an individual’s immune system, enhancing their susceptibility to HPV infections. Benzo[*α*]pyrene, a constitute of tobacco, upregulates the amplification of the HPV genome, thus enhancing the integration of the viral genomes into the host’s genome [62]. The long-term effects of nicotine *in vivo* include the stimulation of the vascular endothelial growth factor, persistent cell proliferation, apoptosis inhibition, suppression of T lymphocyte activity and immunoglobulin levels and increased microvessel density [61].

The hormonal changes observed in an obese individual is positively correlated with the incidence of cervical cancer, whereby females with a body mass index (BMI) over 30 displayed a 2-fold higher vulnerability to cervical adenocarcinoma than individuals with a normal or slightly overweight BMI (≤25) [63]. This results from the conversion of androgen to oestrogen within the peripheral adipose tissue. The additional oestrogen will accelerate cell growth and increase the risk of cancer as observed with oral contraceptives [64].

## Traditional treatment limitations

Cervical cancer treatment is traditionally determined by the stage and extent of the disease. There are essentially three types of standard cervical cancer treatments, including surgery, radiation and chemotherapy. Surgical treatment consists of a radical hysterectomy with pelvic lymphadenectomy (RHL) and conisation for stage 1 and certain stage 2 cervical cancer cases to abrogate the side-effect on fertility [65, 66]. Surgery involves the physical removal of the metastatic tissue from the cervix or cervical canal [66]. Although cervical cancer surgery is minimally invasive, a complete pelvic lymphadenectomy is associated with lymphocele, pelvic nerve impairment and lymphoedema. This anticancer method can cause damage to the sympathetic and parasympathetic branches of the autonomous innervation and blood supply of pelvic organs, leading to colorectal dysfunction [65].

Radiotherapy attempts to destroy cancer cells utilising various forms of radiation including high energy X-rays. Radiotherapy is administered in the advanced phases of stage 1 cervical cancer as a primary, radical curative. This form of therapy consists of brachytherapy, concomitant cisplatin and external beams of radiotherapy [67]. Postoperative, adjuvant radiotherapy aimed to reduce the reoccurrence of cervical cancer is administered to tumours larger than 4 cm with lymph node metastases [68]. Post-radical therapy individuals with adverse histopathologic influences are required to undergo adjuvant pelvic radiation or chemoradiation [69]. Deep cervical stromal invasion, metastatic disease in regional nodes, parametrial extension and positive surgical margins serve as risk factors for the reoccurrence of cervical tumours. Radiotherapy is limited by the induction of vaginal discomfort and dryness, rectal bleeding and stenosis, radiation cystitis, menstrual changes and lymphoedema [70].

Chemoradiation is the preferred method of treatment as it enhances the anticancer properties of radiation. Chemotherapy inhibits the growth of cervical cancer cells by preventing their active division and growth with the aid of chemotherapeutic compounds, such as carboplatin, cisplatin, methotrexate, paclitaxel and topotecan [69, 71]. Although it is able to treat advanced cervical cancer which has spread to subsequent organs, this form of therapy is limited by its detrimental side effects [72]. Several studies have illustrated a higher survival rate in women treated with chemotherapy followed by radiation, as a result of the selection of cross-resistant tumour cells which delay the initiation of the curative therapy [69, 70]. The sensitivity of chemotherapeutic drugs is limited by their inability to distinguish cancer cells from their non-metastatic counterparts; hence, the drugs also inhibit the functioning of healthy cells [71]. Although most side effects are short-term,

such as fatigue, hair loss, nausea and loss of appetite, the long-term effects negatively impact the quality of life of the patients [72]. These include anaemia, neutropenia, thrombocytopenia due to blood marrow cell damage, neuropathy or peripheral neuropathy due to nerve damage (cisplatin and paclitaxel), nephrotoxicity or kidney damage, premature menopause and, in severe cases, infertility [73].

These cervical cancer treatment approaches lack satisfactory outcomes despite the rapid elucidation of biological and the etiological understanding of HPV and cervical cancer. The main limitation lies within their side effects, which, in turn, limits their optimal dosages and efficiency. Hence, it is fundamental to develop novel strategies to treat pre-invasive and invasive cervical cancer [11]. Advances in nanomedicine have the potential to overcome these limitations by improving the management and treatment of cervical cancer. Nanomedicine aims to improve the survival rate of cancer patients by reducing side effects and enhancing the selective delivery of drugs to tumour tissues and uptake of therapeutic compounds, thus increasing antitumour activity [14].

## Nanomedicine

The past decade has elucidated the potential and application of nanotechnology in various aspects of day-to-day life. The use of nanotechnology in pharmaceutical research and development of novel medical approaches has been precluded as a key enabling technology, which enhances the development of innovative medical solutions to address unfulfilled medical needs [74, 75]. The application of nanotechnology for medical purposes has been termed as nanomedicine and is defined as the use of nanomaterials to diagnose, treat, monitor and control diseases [12].

Nanoparticles are classified according to their particle size, surface area and particle size distribution (PSD). Size is the most fundamental characteristic in nanotechnology. Nanoparticles usually range between 1 and 100 nm; however, the size varies with respect to the function, physicochemical and biological characteristics of the nanomaterial utilised [76, 77]. Size is controlled by the ‘bottom-up’ and ‘top-down’ techniques, by which the particle increases to the desired size from an atomic state or is produced by the breakdown of a bulk material into smaller pieces using chemical or mechanical energy [78]. Boverhof *et al*. [78] further reported the importance of surface area (approximately 60 m^2^/cm^3^) and PSD due to the polydispersion of nanomaterials.

Nanomedicine has the potential to overcome the limitations of conventional therapeutic approaches by improving the targeted delivery of therapeutics to tumour sites and enhancing efficiency [13]. Numerous studies have elucidated the potential of nanomedicine in cervical cancer therapy, showing that nanoparticles increased the therapeutic index with reduced side effects by using the passive targeting approach which causes nanocomplex accumulation in tumours [14, 77].

### Medical properties of nanoparticles

Nanomedicine is categorised into three fundamental branches, namely regenerative medicine, nanodiagnosis (diagnosis) and nanotherapy (controlled drug delivery). A fourth emerging category, which combines therapy and diagnostics, termed theranostics displays a promising approach for future cancer therapy [79, 80]. Nanomedicine has revolutionised clinical practices by exploiting multiple mechanisms of effective molecules which could not be utilised to date due to their toxicity, maximising efficacy and reducing harmful side effects, toxicity and required dose [12–14]. Nanoparticles provide a preferential distribution throughout the body by facilitating targeted, controlled and site-specific drug delivery, in addition to improving movement across biological barriers [80].

The compact, small size of nanoparticles offers a high specific surface area with relation to volume, thus resulting in an increase in the surface energy of the particle and making the nanomaterial more reactive [76]. Dynamic changes in the plasma biomolecule adsorption layer surrounding the surface of the nanoparticle, referred to as the ‘corona’, facilitate the transition of the nanomaterial across biological barriers, such as the gastrointestinal and lung fluid, and blood [81, 82]. Nanoparticles also possess variable sizes, shapes, chemical compositions and magnetic, optical and electric properties [83].

The physiopathological nature of the diseases in question and the interaction between the biological fluid of the environment and surface of the nanomaterial must be taken into consideration when designing biologically effective nanoparticles [76, 82]. Understanding the biological processes behind specific diseases enables researchers to design nanomaterials which perform site-specific action on misfolded proteins, mutated genes and infections caused by microorganisms [83]. The interaction between the nanoparticle and the cell is determined by the characterisation of the corona’s biomolecules. This interaction is influenced by the size, surface area, shape, chemistry, energy, porosity, hydrophobicity, presence of ligands and valence and conductance states of the nanomaterials [81, 84]. The intrinsic properties of nanomaterials are highly advantageous in the development of pharmaceutical therapies, including cancer treatment [79].

### Induction of apoptotic pathways for cervical cancer

**Figure 3:**
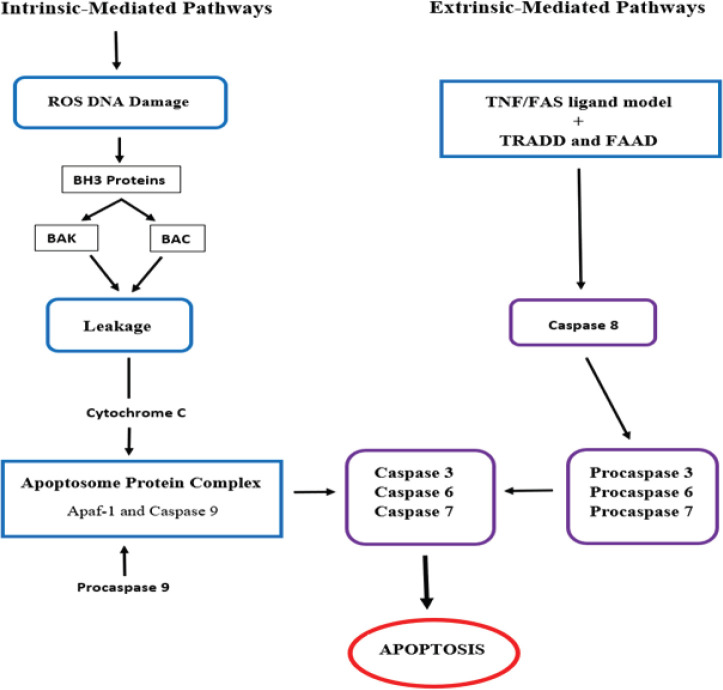
Targeted intrinsic and extrinsic pathways for cervical cancer treatment [11, 85, 87].

Apoptosis is defined as programmed cell death through biochemical mechanisms [85]. Apoptosis is a vital instrument in the functioning and development of the embryo and the immune system, hormone-dependent atrophy and normal cell turnover [86]. In cancer therapy, the apoptosis of metastatic cells can be induced by inhibiting essential cellular pathways. These include intrinsic pathways that target the mitochondria or extrinsic pathways that involve tumour necrosis factor (TNF) receptors and extrinsic signals (Figure 3) [87].

In intrinsic pathways, the p53 protein increases the production of the apoptosis regulator Bcl-2 homologous antagonist (BAK) and bcl-2-like protein 4 (BAX), which, in turn, increases the mitochondrial membrane permeability and the leakage of cytochrome c [85]. Cytochrome c then forms an apoptosome protein complex by binding with apoptotic protease activating factor-1 (Apaf-1), procaspase 9 and adenosine triphosphate. Cleavage of the pro-caspase from the apoptosome produces active caspase 9 proteins, which activate caspase 3 leading to cell death [11]. In extrinsic pathways, apoptosis is induced by a TNF model or first apoptosis signal (Fas) ligand model. The receptors of both these models bind to the TNF receptor-associated death domain and the Fas-associated death domain protein, respectively, to activate caspases 8, which induces the caspase 3 production and cell death [87].

Nanoparticles have been used to deliver anticancer therapeutics which initiate the apoptotic pathway in cancer cells. Nanoparticles coupled with apoptotic pathway stimulators reduce the stimulator’s toxic side effects whilst enhancing cytotoxicity [86]. Silver nanoparticle-bound carboplatin was observed to stabilize the DNA-topoisomerase I, resulting in the accumulation of breaks in the DNA, inhibiting replication and transcription and leading to HeLa cell death at significantly higher toxicity and lower concentrations compared to carboplatin [88].

Targeting mitochondrial apoptotic pathways has also been studied for cervical cancer therapy [11, 87]. The ability of mesoporous silica nanoparticles carrying cytochrome c was shown to effectively induce apoptosis in cervical cancer cells by targeting mitochondrial apoptotic pathways [89]. The cytotoxic and apoptotic effect of silver nanoparticles (AgNPs) in HeLa cells was demonstrated [90]. AgNPs upregulated the mRNA expression of BAX, P53 and caspase 3 in AgNP-treated HeLa cells by targeting the mitochondrial apoptotic pathways. Nanomedicine has revolutionised gene therapy, chemotherapy and photodynamic therapy by improving the delivery of insoluble drugs, circulation, direct targeting or delivery of drugs, delivery of multiple drugs and transcytosis of the drugs across endothelial and epithelial barriers and imaging [12, 91].

#### Gene therapy

Gene therapy modifies the human genome by introducing exogenous genetic material into a cell to treat the underlying cause of a disease [92]. Cancer gene therapy aims to enhance the sensitivity of tumours towards chemotherapeutic drugs and radiation therapies or induces apoptosis by the introduction of suicide or wild-type tumour suppressor genes [93]. Although gene modification has successfully improved cervical cancer therapy through alterations in the cellular signalling pathways, the uptake of genetic cargo by cells is limited [94]. Genetic cargo such as deoxyribonucleic acid (DNA) and ribonucleic acid (RNA) is unable to effectively cross epithelial membranes due to their negative charge, susceptibility to degradation and large size [95], hence the need for suitable nanocarriers of these nucleic acids.

Nanoparticles serve as vehicles by facilitating the binding, condensation and delivery of small interfering RNA (siRNA), messenger RNA (mRNA), microRNA (miRNA) and plasmid DNA (pDNA), to the target sites [96-98]. A targeting ligand is generally attached to a nanocarrier to improve the sensitivity and selectivity of uptake of the genetic cargo by the cancer cells. An increase in transfection efficacy of DNA containing nanoparticles with folic acid moieties over naked DNA was observed in HeLa (cervical cancer) cells [99], whereas efficient luciferase gene silencing using siRNA-selenium nanocomplexes was observed in HeLa-tat-*Luc* cells [98].

#### Chemotherapy

Chemotherapy remains a standard approach to treat cervical cancer. However, the efficiency of conventional chemotherapeutic drugs is limited by their undesirable and severe side effects, lack of specificity, rapid *in vivo* degradation, poor biodistribution, suboptimal concentrations and intervals between treatments [15]. Common cervical cancer chemotherapeutic drugs include cisplatin, paclitaxel and methotrexate. Cisplatin disrupts biological functions and apoptosis of the cancer cells by binding and crosslinking to the DNA of cells. However, this drug has been observed to induce renal failure and neurotoxicity [100]. Other drugs, such as methotrexate, decrease the conversion of dihydrofolates to tetrahydrofolates required for RNA and DNA replication, by inhibiting dihydrofolate reductase and stimulating neutropenia and stomatitis [101].

Nanoparticles can improve the efficacy, bioavailability and distribution of the chemotherapeutic drugs to cancerous tissues whilst reducing side effects and cytotoxicity in healthy tissue, by creating a targeted drug delivery system [88]. Nanoparticles are able to bind and transport drugs across physiological barriers that limit traditional drug delivery systems [16]. The coating of nanoparticles with a polymer layer (e.g., polyethylene glycol) inhibits the binding of proteins *in vivo* and allows the nanoparticles to evade immune responses induced by the reticuloendothelial system and macrophages, which eliminate foreign bodies from the blood system [101].

Silver nanoparticles carrying carboplatin (a platinum-based drug) was shown to increase cytotoxicity in HeLa cells compared to using carboplatin alone, with a further reduction of undesired side effects, improvement in pharmacokinetics and synergism and suppression of drug resistance through distinct mechanisms of action at a low dose [88]. Improved efficiency in cervical cancer treatment using several proteins, polymers and lysosome nanocomplexes containing cisplatin, paclitaxel and methotrexate was also reported [11]. The ability of chitosan and methoxypolyethylene glycol-coated nanoparticles were seen to effectively deliver methotrexate both *in vivo* and *in vitro* and to reduce HeLa cell proliferation [103]. Although polyphenols are promising anti-cervical cancer agents, their clinical application is hindered by their poor solubility and low oral bioavailability [15].

#### Photodynamic therapy

Photodynamic therapy (PDT) induces apoptosis of cancerous cells using light-sensitive compounds that react with biological compounds under selective lighting [14]. As a minimally invasive anticancer therapy method, photodynamics is considered as a promising modality for the ablation of tumour cells in a variety of cancers, including cervical cancer [14]. PDT involves the use of a non-toxic near-infrared light (620 nm and 850 nm) which has maximum tissue permeability and a photosensitizer to improve anticancer effects [99]. Exposure to light stimulates the activation of the photosensitizers which are reduced to a photosensitizers’ triplet, which further reacts with oxygen to produce reactive oxygen species. These cytotoxic molecules induce a series of biological reactions which eventually lead to the death of the cancer cells [102].

Most photosensitizers occur naturally in plant extracts such as hypericin (*H. perforatum*, 514–593 nm), thiophenes (*E. latifolius Tausch*, 225 and 400 nm) and curcumin (*Curcuma longa*, 350 to 450 nm). Although these extracts are capable of treating cancer cells, the compounds have poor solubility properties and are ineffective in clinical applications [103]. Lipid nanocapsules embedded with hypericin were found to improve the solubility and efficiency of the compound in HeLa cells [104].

Gold nanoparticles have shown potential in the transport of protoporphyrin IX, a heterocyclic organic photosensitive compound, to HeLa cells [14]. These gold nanoparticles increased cytotoxicity and produced higher reactive oxygen species by absorbing radiation at 630 nm. Furthermore, gold nanoparticle-mediated 2-mercapto-5-nitro benzimidazole (MNBI) was also reported to increase cytotoxicity in cervical cancer cells. MNBI produces nitric oxide (NO), which is toxic to healthy cells; however, the MNBI nanocomplex mediated delivery directly to the HeLa cells minimising harmful side effects on healthy cells [105].

### Dual-role nanoparticles

Nanoparticles often enhance cancer therapy by serving a dual role, whereby the particle can carry as well as co-deliver multiple anticancer drugs, including genetic material, photosensitising molecules, proteins and chemotherapeutic drugs. Polymeric nanoparticles which co-delivered siRNA and cisplatin were shown to effectively decrease the viability of cervical cancer cell lines *in vitro* [106]. PLGA magnetic nanoparticles were also used to co-deliver curcumin and 5-fluorouracil to cancer cells [107], whereas methotrexate and 5-fluorouracil-loaded layered double hydroxide produced enhanced cytotoxicity in cervical cancer cells compared to the drugs alone [108]. The potential of a siRNA, anti-MCL 1 and anti-E7 cocktail against oncoproteins E6 and E7 and anti-apoptotic protein MCL-1 against HPV-related cervical cancer was also noted [109]. Besides carrying and delivering therapeutics, some nanoparticles themselves exhibit anticancer properties.

The concept of gold nanoparticles was envisaged over 100 years ago by Faraday and are popular in nanomedicine [110]. They have been utilised as delivery vehicles for anticancer therapeutics due to their favourable and unique characteristics which include biocompatibility, small size and dispersive shapes, strong surface plasmon resonance and high dispersity. They also display antibacterial properties [90], as well as antiproliferative properties towards certain cancer cell lines [107]. The anticancer properties of gold nanoparticles in HeLa cells have been reported [110]. Silver nanoparticles have also illustrated antibacterial and anticancer properties by targeting and inhibiting the nuclear DNA-dependent serine/threonine-protein kinase (DNA-PKcs) and c-Jun N-terminal kinases causing extensive damage to the telomeres in cancer cells [21]. Selenium nanoparticles possess antioxidant and cytotoxic activity in cancer cells, high bioavailability and low toxicity, in addition to serving as delivery vehicles for anticancer compounds [111]. Nanoparticles have also shown their potential for combination therapy further enhancing their anticancer potential in the treatment of cervical cancer.

## Conclusion

Cervical cancer remains a global health crisis with several common and unavoidable risk factors. Despite the rapid elucidation of the biological and etiological understanding of HPV and cervical cancer, traditional treatments, such as surgery, radiation and chemotherapy, lack satisfactory outcomes due to numerous limitations. Nanomedicine has revolutionised and can extensively improve cervical cancer therapy by enhancing the targeting of apoptotic pathways. The low toxicity, biocompatibility, photosensory and anticancer properties of nanoparticles, together with their potential for dual treatment by acting synergistically with its payload, make them ideal platforms for cervical cancer treatment. This review serves to enhance the understanding and need for these non-toxic, safe, stable delivery systems, which are capable of protecting their therapeutic cargo against degradation and offering high therapeutic indices. The reduced cost implications in particular will be attractive to low-income and developing countries. However, nanomedicine in cervical cancer therapy is still in its infancy, and further clinical studies need to be undertaken to fully appreciate its benefits.

## Conflicts of interest

The authors declare that they have no conflicts of interest.
